# Supplementary material for: Triage of Critically Ill Patients: Characteristics and Outcomes of Patients Refused as Too Well for Intensive Care
Source: J Clin Med. 2023 Aug 25;12(17):5513. doi: 10.3390/jcm12175513 (PMC10488145; doi:10.3390/jcm12175513)
Supplement: Supplementary file 1 [file jcm-12-05513-s001.zip › Manuscript.V02.b.Suppl.Table_S1.pdf]

Journal of Clinical Medicine

MDPI

Table S1. Data collected at baseline

	Admitted	Refused
**Patient characteristics**		
Age	x	x
Sex	x	x
Diagnosis for ICU request	x	x
Metastatic or haematological malignancy	x	x
Limitations of life-sustaining treatments	x	x
Time since hospital admission	x	x
SOFA score [1]		x
**Organisational factors**		
Surgical status	x	x
Location at time of ICU demand	x	x
Time of demand for ICU admission	x	x
Availability of ICU beds at time of ICU demand	x	x
**Modalities for ICU consultation**		
ICU Physician's grade of experience		x
Written documentation of ICU refusal		x
Duration of consultation		x
Type of consultation (bedside, phone, chart)		x

[1] The Sequential Organ Failure Assessment (SOFA) score was assessed by using all parameters available at time of request.
ICU Intensive care unit
