# Supplementary material for: Triage of Critically Ill Patients: Characteristics and Outcomes of Patients Refused as Too Well for Intensive Care
Source: J Clin Med. 2023 Aug 25;12(17):5513. doi: 10.3390/jcm12175513 (PMC10488145; doi:10.3390/jcm12175513)
Supplement: Supplementary file 1 [file jcm-12-05513-s001.zip › Manuscript.V02.b.Suppl.Table_S2.pdf]

Journal of
Clinical Medicine

Table S2. Organ specific SOFA scores of patients deemed too well for ICU according to event occurrence

	Eventless evolution (N = 215)	Unexpected event [1] (N = 16)	p value
**SOFA Score, specific organ dysfunction [2]**			
Neurologic failure			1.00
Neuro-SOFA >1	3 (1.4)	0 (0.0)	
Neuro-SOFA <=1	212 (98.6)	16 (100.0)	
Respiratory failure			0.03
Resp-SOFA >1	48 (22.3)	8 (50.0)	
Resp-SOFA <=1	167 (77.7)	8 (50.0)	
Cardio-vascular failure			n.a.
CV-SOFA >1	0 (0.0)	0 (0.0)	
CV-SOFA <=1	215 (100.0)	16 (100.0)	
Renal failure			0.39
Renal-SOFA >1	22 (10.2)	3 (18.8)	
Renal-SOFA <=1	193 (89.8)	13 (81.2)	
Liver failure			1.00
Digest-SOFA >1	6 (2.8)	0 (0.0)	
Digest-SOFA <=1	209 (97.2)	16 (100.0)	
Haematologic failure			< 0.01
Haemato-SOFA >1	7 (3.3)	7 (43.8)	
Haemato-SOFA <=1	208 (96.7)	9 (56.2)	
SOFA Score, number of organs with > 1 point			< 0.01
0	139 (64.6)	2 (12.5)	
1 - 2	75 (34.9)	14 (87.5)	
3 - 6	1 (0.5)	0 (0.0)	

[1] An unexpected event was defined as either ICU admission without evidence of another indication, or death without treatment limitations for ICU, within 7 days after ICU refusal.
[2] Organ dysfunctions were defined as a SOFA score of 2 or higher for each component.
